# MicroRNAs' Involvement in Osteoarthritis and the Prospects for Treatments

**DOI:** 10.1155/2015/236179

**Published:** 2015-10-26

**Authors:** Xiao-Ming Yu, Hao-Ye Meng, Xue-Ling Yuan, Yu Wang, Quan-Yi Guo, Jiang Peng, Ai-Yuan Wang, Shi-Bi Lu

**Affiliations:** Institute of Orthopedics, Chinese PLA General Hospital, Beijing 100853, China

## Abstract

Osteoarthritis (OA) is a chronic disease and its etiology is complex. With increasing OA incidence, more and more people are facing heavy financial and social burdens from the disease. Genetics-related aspects of OA pathogenesis are not well understood. Recent reports have examined the molecular mechanisms and genes related to OA. It has been realized that genetic changes in articular cartilage and bone may contribute to OA's development. Osteoclasts, osteoblasts, osteocytes, and chondrocytes in joints must express appropriate genes to achieve tissue homeostasis, and errors in this can cause OA. MicroRNAs (miRNAs) are small noncoding RNAs that have been discovered to be overarching regulators of gene expression. Their ability to repress many target genes and their target-binding specificity indicate a complex network of interactions, which is still being defined. Many studies have focused on the role of miRNAs in bone and cartilage and have identified numbers of miRNAs that play important roles in regulating bone and cartilage homeostasis. Those miRNAs may also be involved in the pathology of OA, which is the focus of this review. Future studies on the role of miRNAs in OA will provide important clues leading to a better understanding of the mechanism(s) of OA and, more particularly, to the development of therapeutic targets for OA.

## 1. Introduction

OA is a joint disease of relatively frequent occurrence that is characterized by progressive damage to the articular cartilage and subchondral bone, causing pain and disability in older adults [1, 2]. Common features of OA include joint pain, turgidity, synovial inflammation, and loss of function. The most common risk factors for OA include age, gender, obesity, genetic predisposition, prior joint trauma, mechanical factors, malformation, and hypoplasia. Several tissues of the joint, including the cartilage, synovial membrane, and subchondral bone, play significant roles in the development of OA pathology. OA is associated with degradation of cartilage, ultimately leading to the complete loss of cartilage, due to degeneration and then apoptosis of chondrocytes, loss of bone, with increased bone remodeling followed by slow reversal. Alterations beneath the cartilage at the osteochondral junction have drawn interest as mediators of the structural progression of OA. Osteochondral alterations happen early during the development of OA and can aggravate the pathological changes elsewhere in the joint [3]. In this process, inflammatory factors have a significant effect, but genetic factors may be the biggest issue. Chondrocytes and bone cells must express appropriate genes to achieve joint homeostasis and if this is upset, this can lead to OA. The genes expressed and the transcription factors that control chondrogenesis and osteogenesis have been widely studied.

MicroRNAs (miRNAs) are a large family of small (21–25-nucleotide) noncoding RNAs. There have been many publications describing miRNA synthesis and function, and miRNAs have emerged as important posttranscriptional regulators of gene expression that function by binding to specific sequences within target mRNAs. Each miRNA recognizes target sequences in many genes and each gene contains target sequences for the binding of many miRNAs, leading to translational repression or mRNA degradation. miRNAs regulate gene expression by binding to the 3′-untranslated region (UTR) of their target mRNAs. Many miRNAs exhibit a tissue-specific or developmental stage-specific expression pattern, which has been reported to be associated with diseases, such as cancer, leukemia, viral infections, and bone disease [4–7]. Recent evidence indicates that miRNAs have important roles in regulating osteogenic and chondrogenic differentiation and proliferation, eventually influencing the catabolism and anabolism of bone and cartilage. This review focuses on miRNA in OA. Recently, miRNA has been described as a novel therapeutic strategy for OA and is expected to provide targets for clinical treatment and biomarkers for diagnosis and prognosis for OA [8]. Thus, a more complete understanding of all facets of miRNA function in the joint is necessary.

## 2. MicroRNA Formation and Biological Function

**Figure 1:**
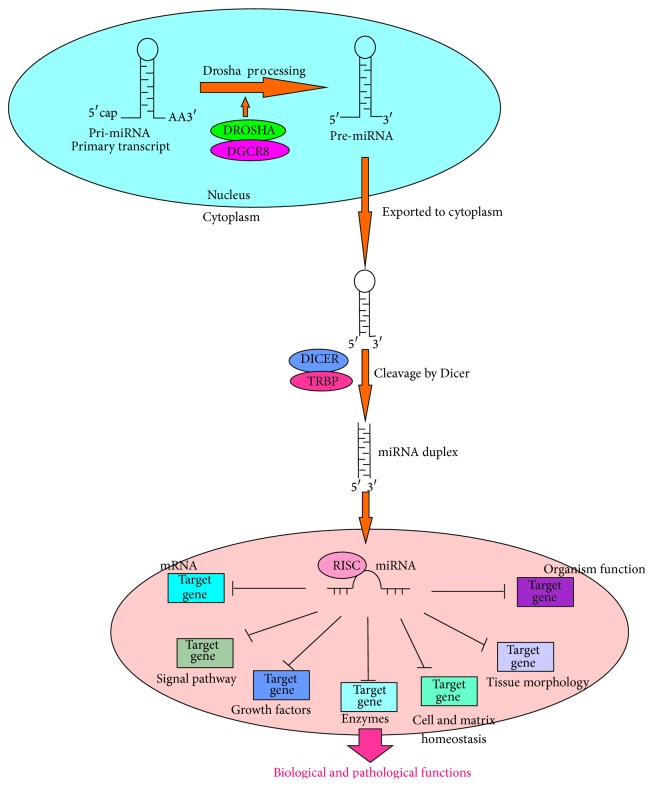
MicroRNA function. Pathways to generate miRNAs. Multiple mRNAs and biological pathways can be targeted by miRNAs. Molecular networks can be regulated by miRNAs, including various aspects of biological and pathological functions.

Many miRNAs are located within introns of protein-coding genes, and a small proportion originate from exons or noncoding mRNA-like regions [9]. Despite the distinct biological differences between miRNAs and mRNAs, present evidence suggests that their transcripts share common mechanisms of transcriptional regulation [10]. miRNAs are transcribed by RNA polymerase II and processed in the nucleus by a protein complex that contains RNase III, Drosha, and DGCR8 (Figure 1). Exportin 5, a GTP-dependent and dsRNA-binding protein, mediates the process that transports the resulting 60–80-nucleotide pre-miRNA cleavage product to the cytoplasm. Another protein complex, Dicer, in the cytoplasm, containing the RNase III enzyme, cleaves pre-miRNA at the stem-loop structure to generate an approximately 22-nucleotide imperfect double-stranded miRNA duplex [11]. One strand of the duplex is designated as the guide miRNA and remains stably associated with the miRNA-induced silencing complex (mRISC). The other strand, known as the passenger strand, can be degraded rapidly. It is possible that both strands could be used differently in relation to extracellular or intracellular effects, indicating that the two strands may regulate a more diverse set of protein-coding genes as needed, or the selection of strand could be tissue-specific. Mature miRNA guides the mRISCs to the 3′-UTR of its target mRNA, which is partially or fully complementary to the seed sequence of the mature miRNA [12]. Then, miRNA binding to the 3′-UTR can promote or repress the translation of the target mRNA (Figure 1). This may be an evolutionary strategy to diversify miRNA-based gene silencing.

## 3. Role of miRNA in Bone Development

The precise role and expression of miRNA in bone development have been reported and established.

### 3.1. Regulatory Activity of miRNAs in Osteoblasts

#### 3.1.1. Promotion

Some miRNAs stimulate osteoblastogenesis (Table 1). Osterix (Osx) is an osteoblast-specific transcription factor that is essential for bone formation [71]. Osx directly or indirectly downregulates expression of miR-133a and miR-204/211 and upregulated expression of miR-141/200a to maintain appropriate levels of Runx2, Sclerostin, ALP, and Dlx5 proteins for the optimal differentiation and function of osteoblasts [13]. miR-214 is a novel regulator of Osx and suppresses the osteogenic differentiation of C2C12 myoblast cells, providing novel insights into the roles of miRNAs in osteoblast differentiation [27]. BMPs and RUNX2 are key signaling genes inducing osteoblastogenesis. Many miRNAs can induce osteoblastogenesis by indirectly enhancing the expression or activity of BMPs and RUNX2. miR-2861 promotes osteoblast differentiation by repressing histone deacetylase 5 (HDAC5) expression at the posttranscriptional level, which was found in ST2 stromal cells during BMP2-induced osteogenesis [14]. miR-15b induces osteoblast differentiation of human mesenchymal stromal cells, targeting BMP-binding endothelial regulator (BMPER), a BMP inhibitor [15]. miR-1228 inhibits BMP-2 kinase (BMP2K) translation to induce 1,25-dihydroxyvitamin D-induced osteoblastogenesis of osteoblasts [16]. miR-764-5p could inhibit CHIP/STUB1 translation, a protein that promotes Runx2 protein degradation, and induced osteoblast differentiation of mouse MC3T3-E1 cells [17].

miRNAs also regulate another significant signaling pathway, the Wnt signaling pathway, to affect osteoblastogenesis. Zhang et al. investigated how miR-335-5p decreases Dickkopf-related protein 1 (DKK1) expression to activate Wnt signaling and promote osteogenic differentiation [18]. miR-218 stimulates the Wnt pathway by downregulating three inhibitors of Wnt signaling, SOST, DKK2, and SFRP2, during the process of osteogenesis to enhance osteoblast differentiation [19]. Also, miR-29a was found to target osteonectin, an inhibitor of Wnt signaling, to enhance osteoblastogenesis in mouse MC3T3 cells [20]. Both miR-142-3p and miR-27 can promote osteoblastogenesis and indirectly enhance Wnt signaling by inhibiting adenomatous polyposis coli (APC) expression [21].

In addition, miRNAs promote osteoblastogenesis by affecting other signaling factors. miR-210 acts as a positive regulator of osteoblast differentiation and suppresses the TGF-*β*/activin signaling pathway through inhibition of ACVR1B [23]. miR-26a modulates late osteoblast differentiation by targeting the SMAD1 transcription factor [24]. miR-96 simulates osteoblast differentiation of hMSCs by increasing the expression of FABP4, and miR-199a can promote osteoblast differentiation of hMSCs by downregulating the expression of transcription factor SOX9 and upregulating the expression of* aggrecan* [25].

#### 3.1.2. Inhibition

miRNAs have also been demonstrated to have inhibitory roles in osteoblastogenesis (Table 1). Osx was shown to be a direct target of miR-637, suggesting that miR-637 could suppress osteoblast differentiation through direct suppression of Osx expression in hMSCs [29]. Inhibition of the BMP/RUNX2 signaling pathway is another common route for miRNAs to prevent osteoblastogenesis. For example, miR-133 targets Runx2 directly, and miR-135 targets Smad5 directly to inhibit BMP2-induced osteogenic differentiation [30]. miR-206 inhibits osteoblast differentiation by suppressing the expression of Cx43, a major gap junction protein in osteoblasts [31]. miR-100 has been suggested to play a negative role in osteogenic differentiation by targeting BMPR2 directly [32]. miR-155 was demonstrated to be involved in TNF-*α*-mediated inhibition of osteogenesis differentiation, which targets a suppressor of cytokine signaling 1 (SOCS1) directly, affecting BMP-2-induced osteoblast differentiation [33]. miR-370 inhibits BMP-2-induced preosteoblast differentiation in regulating the expression of BMP-2 and Ets1 [34]. miR-433 inhibits RUNX2 and ALP expression, suppressing BMP-2-induced osteoblast differentiation [36]. miR-30 family members (miR-30a, miR-30b, miR-30c, and miR-30d) can inhibit osteogenesis by targeting Smad1 and RUNX2 [37].

Inhibition of the Wnt signaling pathway can also impede osteoblastogenesis. Mizuno et al. showed that miR-221 and miR-1274a inhibited osteoblast differentiation in unrestricted somatic stem cells (hUSSCs) by suppressing Wnt signaling [39].

miRNAs can also affect other genes, independently of BMP/RUNX2 and Wnt signaling pathways, to inhibit osteoblastogenesis. miR-214 can inhibit osteoblast activity and matrix mineralization, by targeting ATF4 [38]. Kim et al. described the role of miR-125b as an inhibitor of osteoblastic differentiation by downregulating cell proliferation [40]. miR-182 functions as a FoxO1 inhibitor, antagonizing osteoblastogenesis [41]. miR-138 inhibits osteogenic differentiation of hMSCs by suppressing expression of PTK2 [42]. Kahai et al. showed miR-26a to be an inhibitor of osteoblast differentiation, targeting Smad1 transcription [43]. miR-378 impedes osteoblastogenesis by preventing the expression of GalNAc-t7 (UDP-N-acetyl-alpha-D-galactosamine: polypeptide-acetylgalactosaminyltransferase 7) [44].

### 3.2. Regulatory Activity of miRNAs in Osteoclasts

#### 3.2.1. Promotion

Certain miRNAs are known that can affect the proliferation and differentiation of osteoclasts (Table 1). miR-21 was identified as an inhibitor of programmed cell death 4 (PDCD4) in RANKL-induced osteoclastogenesis, rescuing osteoclast development [44]. miR-155 can stimulate osteoclast activity by targeting SHIP, a suppressor of osteoclastogenesis [45]. miR-34c can enhance osteoclast differentiation by regulating the Notch signaling pathway, including Notch1, Notch2, and Jag1 in a direct manner [46]. miR-223 stimulates osteoclast differentiation and function via suppressing NF1-A expression [47]. miR-378 can be upregulated when osteoclast differentiation is induced, and it promotes cell survival [48].

#### 3.2.2. Inhibition

miRNAs also can inhibit osteoclast differentiation (Table 1). Rossi et al. reported that miR-29b can decrease human osteoclast differentiation by targeting c-Fos and MMP-2 [49]. miR-223 can suppress osteoclastogenesis if it is overexpressed [72]. miR-146a can target TNF receptor-associated factor-6 (TRAF6) to inhibit osteoclastogenesis in human peripheral blood mononuclear cells (PBMCs) [50]. miR-155 can also impede osteoblastogenesis by suppressing the expression of MITF [73].

Interestingly, some miRNAs regulating osteoblastogenesis are also involved in osteoclastogenesis. For example, miR-378 [43] inhibited osteoblast differentiation while also regulating osteoclast differentiation [48]. miR-29b inhibits osteoclastogenesis and induces osteoblastogenesis [49]. miR-155 regulates osteoclastogenesis [73] but also inhibits osteoblastogenesis [32]. However, these “shared” miRNAs operating in both osteoblastogenesis and osteoblastogenesis target different genes and signaling pathways in osteoblasts and osteoclasts, indicating the different mechanisms of the same miRNAs in regulating osteoblastogenesis and osteoclastogenesis.

In consideration of the previous studies, we know that change in the subchondral bone is one of the factors of OA, and when the functions of these miRNAs mutated in osteoblasts or osteoclasts, they can result in the changes in the subchondral bone, which may induce the progression of OA.

## 4. Role of miRNAs in Chondrogenesis

Dicer is an essential component for miRNA biogenesis, a deficiency of which in chondrocytes can lead to a reduction in the number of proliferating chondrocytes via accelerated differentiation and decreased proliferation into hypertrophic chondrocytes [74, 75]. Recently, the important role of miRNAs in chondrogenesis and cartilage homeostasis has received much attention (Table 1).

### 4.1. Promotion

Chondrogenesis in mouse MSCs leads to upregulation of miR-124a and miR-199a [52]. miR-140 and miR-30a are upregulated during chondrogenesis [53]. Chondrogenesis can also result in the upregulation of miR-130b, miR-152, miR-28, miR-26b, and miR-193b [54]. Zhang et al. found 12 miRNAs that were differentially expressed before or after chondrogenic induction: miR-193b, miR-199a-3p/has-miR-199b-3p miR-455-3p, miR-210, miR-381, miR-92a, miR-320c, and miR-136 were upregulated while miR-490-5p, miR-4287, miR-BART8, and miR-US25-1 were downregulated. This group of miRNAs may play important roles in regulating chondrogenesis differentiation of HADSCs [55].

### 4.2. Inhibition

miR-96 is downregulated in chondrogenesis in mouse MSCs [52]. Two miRNA clusters, miR-143/miR-145 and miR-132/miR-212, are downregulated during chondrogenesis [53]. miR-145 negatively regulates chondrogenesis by targeting Sox9 transcription [56]. miR-9 was shown to inhibit the survival of chondroblasts and articular chondrocytes during chondrogenesis by targeting protogenin, overexpression of which can stimulate the activation of caspase-3 signaling and increased apoptosis [57]. miR-21 could attenuate the process of chondrogenesis by targeting growth differentiation factor 5 (GDF-5) directly [58]. miR-337 is a repressor of TGFBR2 and is involved directly in chondrogenesis [59].

## 5. miRNAs in Articular Cartilage

Articular cartilage provides an enduring friction-free tissue for the movement of limbs. This significant function of articular cartilage is achieved by a cartilage-specific extracellular matrix (ECM). Aggrecans and type II collagen are two prime components of the ECM, and MMPs, which include matrix-degrading enzymes, and ADAMTSs are associated with the degradation of ECM. The balance of these catabolic and anabolic processes is essential for cartilage homeostasis. Many miRNAs have been recognized as vital factors for the development, maintenance, and destruction of articular cartilage.

Miyaki et al. showed that miR-140 regulated cartilage development and homeostasis, and it can be reduced in OA cartilage and in response to IL-1*β*, resulting from a chondrocyte differentiation-related expression pattern, and this reduction may contribute to abnormal gene expression in OA [64]. miR-27b can inhibit the IL-1*β*-induced expression of MMP-13 protein in chondrocytes to reduce the degradation of cartilage [60]. Díaz-Prado et al. observed that seven miRNAs were differentially expressed in normal and OA chondrocytes [61]. Among them, miR-483-5p was upregulated in OA chondrocytes, and miR-149, miR-582-3P, miR-1227, miR-634, miR-576-5p, and miR-641 were downregulated in OA chondrocytes but upregulated in normal chondrocytes, indicating that these seven miRNAs were involved in the development, maintenance, and destruction of articular cartilage. miR-125b can target ADAMTS-4 to inhibit normal chondrocyte ECM degradation [66]. miR-127-5p inhibits normal cartilage degradation via suppressing IL-1*β*-induced MMP-13 production [67]. miR-146a increases the levels of VEGF and damages the TGF-*β* signaling pathway by targeting inhibition of Smad4, contributing to the mechanical injury and apoptosis of chondrocytes [62]. miR-199a can directly prevent the activity of cyclooxygenase-2 (COX-2) and inhibit IL-1*β*-induced COX-2 protein expression in chondrocytes [68]. miR-558 directly suppresses the activity of COX-2 mRNA and inhibits IL-1*β*-induced catabolic effects in chondrocytes to protect the homeostasis of cartilage [69]. miR-675 can upregulate ECM molecules, such as type II collagen [70]. Yang et al. [56] and Martinez-Sanchez et al. [63] found that overexpression of miR-145 suppressed SOX9 in chondrocytes, and expression of matrix genes in articular cartilage—COL2A1, ACAN, COMP, COL9A2, and COL11A1—was inhibited and that of hypertrophic phenotype genes—RUNX2 and MMP-13—was stimulated.

## 6. Role of miRNAs in OA

**Figure 2:**
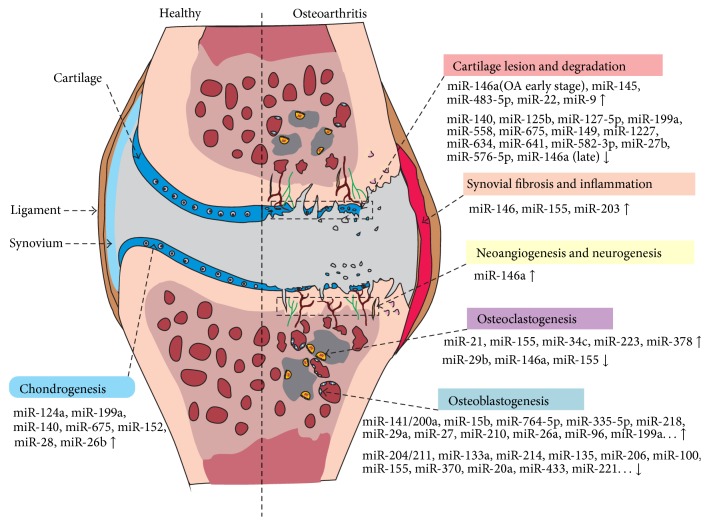
Differential expression of miRNAs between normal and osteoarthritic joints. Changes in miRNA expression can lead to the destruction of joint homeostasis, involving alterations in chondrogenesis, cartilage degradation, synovial inflammation, neurogenesis, osteoblastogenesis, and osteoclastogenesis. miR-146a is involved in cartilage degradation, synovial inflammation, neoangiogenesis, and osteoclastogenesis.

Osteoarthritis is one of the most common chronic diseases; it results in the chondrocyte phenotype and changes in cartilage homeostasis, as well as metabolic changes in subchondral bone, which is an integral part and active component of the OA disease process [76]. The joint disorder is caused mostly by excessive mechanical loading and movement; ultimately, cartilage and bone receive the stress and dissipate it simultaneously. Thus, the cartilage and bone are continuously challenged biomechanically. It is accepted that the balance between catabolic and anabolic rates in bone and cartilage, including the synthesis and degradation of cartilage, is perturbed in OA. Alterations in differentiation, proliferation, activity, and apoptosis of bone cells and chondrocytes are also associated with the development of OA [1]. Recently, several studies have shown that miRNAs play important roles in these processes in OA (Figure 2). Moreover, various correlations between miRNAs and pathological conditions in bone and cartilage add more complexity in OA development.

Iliopoulos et al. tested the expression of 365 miRNAs using microarrays and identified 16 that were expressed differentially in OA cartilage versus normal controls: seven miRNAs (miR-29a, miR-140, miR-25, miR-337, miR-210, miR-26a, and miR-373) were downregulated and 9 (miR-483, miR-22, miR-377, miR-103, miR-16, miR-223, miR-30b, miR-23b, and miR-509) were upregulated [77]. Jones et al. also investigated the expression of 157 miRNAs and showed that 17 were differentially expressed, with 4-fold variation or more when comparing normal and late-stage OA cartilage [78]. Díaz-Prado et al. analyzed 723 miRNAs and identified seven that were differentially expressed in normal and OA human chondrocytes: miR-483-5p was upregulated and miR-149-3p, miR-582-3p, miR-1227, miR-634, miR-576-5p, and miR-641 were downregulated in OA chondrocytes [61]. miRNA-140 was expressed at a lower level in OA cartilage than in normal cartilage [64]. miRNA-27a expression was found to be reduced in OA versus normal chondrocytes [64]. miRNA-146a was highly expressed in the early stages of OA cartilage and decreased in advanced stages of OA [79].

### 6.1. miRNAs Related to Aging

Age is an important risk factor for the development of OA, making the joint more vulnerable to OA risk factors, due to age-related dysregulation of matrix, and catabolic and anabolic processes in cartilage and bone generally. Although the relationship between miRNAs and aging is not fully understood, many studies have provided evidence to demonstrate that miRNAs are associated with aging. Miyaki et al. showed that miR-140 was a regulator of cartilage development and homeostasis, loss of which could contribute to the development of age-related OA [65]. Ukai et al. showed that miR-199a-3p and miR-193b were upregulated with age and associated with Col-II, aggrecan, and SOX9 downregulation, and miR-320c was downregulated with aging and associated with ADAMTS5 upregulation, indicating that miR-199a-3p and miR-193b were involved in senescent chondrocytes, and miR-320c was involved in the juvenile chondrocytes in regulating cartilage metabolism [80]. miR-21 has also been shown to increase in aging cartilage [81].

### 6.2. miRNAs Related to Mechanical Loading

Appropriate mechanical stress is required for cartilage and bone homeostasis. However, excessive impact energy or chronic significant mechanical loading can lead to chondrocyte damage and OA. Guan et al. identified that miR-365 was necessary for the mechanical stimulation of chondrocyte proliferation under cyclic loading conditions; this was shown in culturing primary chicken chondrocytes in a three-dimensional collagen scaffold as a mechanoresponsive miRNA, directly targeting HDAC4 and stimulating RUNX2 and Ihh (Indian hedgehog) expression [82]. Dunn et al. showed that miR-222 expression in the weight-bearing anterior medial condyle was higher than that in the posterior non-weight-bearing medial condyle in the articular cartilage [83], indicating that miR-222 is a potential regulator of the articular cartilage mechanotransduction pathway. miR-146a is overexpressed in the mechanical injury of human chondrocytes, increasing the levels of VEGF and inhibiting Smad4 to damage the TGF-*β* signaling pathway, contributing to the pathogenesis of OA [62]. Thus, the identification of mechanisms of regulation by miRNAs in the mechanical loading of cartilage is important for developing effective treatments for OA.

### 6.3. miRNAs Related to Pain

The joint degradation associated with OA is a common phenomenon and causes chronic and severe pain, especially in the knee joints. As a key debilitating symptom of OA, pain is associated with many changes in gene expression in damaged peripheral tissues and neurons. It has been reported that chronic pain in OA is caused by inflammatory responses that modulate gene expression [84]. Li et al. found that miR-146a regulated knee joint homeostasis and OA-related algesia by balancing inflammation in the cartilage and synovium with pain-related factors in glial cells [85]. miR-146a and/or the miR-183 clusters have also been identified as being closely associated with the stimulation of inflammatory pain mediators and could be a powerful therapeutic target for OA [86].

### 6.4. miRNAs Related to Inflammation

Osteoarthritis is a complex disease in which inflammation plays a significant role, pertaining to development and progression, as a result of inflammatory mediators such as cytokines and prostaglandins released by cartilage, bone, and synovium, involving soluble mediators from local or systemic sources. Recently, some miRNAs have emerged as important controllers of Toll-like receptor (TLR) pathways, which can activate NF-*κ*B and induce the expression of many genes in establishing the inflammatory response in OA and of the proinflammatory cytokine IL-1. miR-155 has a negative effect on the TLR/IL-1 inflammatory pathway by preventing the activation of TGF-*β*-activated kinase 1 and NF-*κ*B and MAPK [87]. miR-146a inhibits the activation of the TLR/IL-1 inflammatory pathway through targeting IL-1 receptor-associated kinase 1 and TNF receptor-associated factor-6 [88]. Interestingly, the expression of miR-146a is elevated markedly by IL-1*β* and TNF-*α* stimulation [89], suggesting a feedback loop involving miR-146a and TLR signaling. Several miRNAs have been reported to be induced by IL-1*β*, such as miR-34a [90], miR194 [91], and miR-27b [60]. Expression of miR-34a was significantly induced by IL-1*β*, promoting chondrocyte apoptosis, and downregulated the expression of type II collagen [90]. miR-27b regulates MMP-13 expression by interacting with the 3′-UTR of MMP-13 mRNA at the posttranscriptional level [60]. miR-140 was identified as being suppressed by IL-1*β* [64], and the expression of miR-140 was found to be NF-*κ*B-dependent [92]. miR-101 participates in IL-1*β*-induced chondrocyte ECM degradation, and IL-1*β*-induced ECM degradation can be prevented when miR-101 is downregulated [93]. miRNA-210 can decrease inflammation in joint cavity in OA, by targeting the death receptor 6 (DR6) and inhibiting NF-*κ*B signaling pathway [94]. It was found that miRNA-130a regulates an underlying mechanism of OA, by regulating the expression of TNF-*α*, and it can be a novel target in OA [95]. miRNA-25 can induce COX-2 overexpressed since miRNA-25 is upregulated by WFA, which can influence the WFA-induced inflammation in OA [96]. It was proved that miRNA-149 is downregulated in OA chondrocytes, which seems to be correlated to overexpression of TNF-*α*, IL-1*β*, and IL-6 [97]. Onju Ham et al. showed that inhibition of protein kinase A (PKA) signaling in synovial fluid-derived mesenchymal stem cells (SFMSCs) using miRNA-23b can be a useful method for the treatment of degenerative arthritis [98]. miRNA-9, miRNA-98, and miRNA-146 were identified by functional analysis in primary chondrocytes that they can regulate the expression of TNF-*α*, and miRNA-9 was upregulated in OA tissue and can inhibit secretion of MMP-13 in chondrocytes [99]. miRNA-488 inhibited MMP-13 activity to promote chondrocyte differentiation/cartilage development through targeting ZIP-8, and suppression of ZIP-8 in OA can reduce cartilage degradation [100].

## 7. miRNAs in the Diagnosis and Therapy of OA

### 7.1. Diagnosis

Although it is still unclear whether measurement of miRNAs in serum may be a useful tool for the diagnosis of OA, we believe that miRNAs can be powerful diagnostic biomarkers for the development of OA; extracellular miRNAs can be detected in almost all body tissues and fluids [101, 102]. miR-21 was the first discovered miRNA biomarker in serum [103]. Subsequently, it has been reported that the serum miRNAs are useful for the diagnosis, and determining the prognosis, of solid cancers and leukemia [102]. Murata et al. found many miRNAs in plasma, and some of them differed significantly between OA and normal tissues [104]. The measurement of miRNAs in peripheral blood mononuclear cells (PBMCs) is also useful in developing a biomarker for OA. If a subject is suffering from OA, circulating PBMCs could accumulate in the synovium and produce proinflammatory cytokines and proteinases associated with OA progression. The high expression of miR-155, miR-146A, miR-181a, and miR-223 in PBMCs from OA patients, compared with normal controls, may be connected with the pathogenesis of OA [105].

### 7.2. Therapy

There has been more than 100 clinical trials worldwide based on miRNA regulation to treat diseases, including cancers and cardiovascular condition, but none for OA yet [106]. On the basis of the important roles of miRNAs in bone and cartilage homeostasis, there could be a novel therapeutic opportunity for the treatment of OA by targeting the expression and activity of miRNAs. It has been reported that the silencing of miR-34a by locked nucleic acid- (LNA-) modified antisense oligonucleotides could effectively reduce IL-1*β*-induced chondrocyte apoptosis [90]. Nagata et al. showed an intra-articular injection of synthetic double-stranded miR-15a resulted in cell apoptosis through preventing action of the target gene BCL-2 in the synovium of osteoarthritic mice. Labeled miR-15a that was injected into the articular cavity was detected in synovium cells, but not in chondrocytes [107]. It has been demonstrated that miR-146a regulates cytokine signaling through a negative feedback loop, and miR-140 regulates cartilage homeostasis. These findings suggest that the administration of miR-146a and miR-140 might be candidate targets for new treatments for the early stage of OA and that several miRNAs combined could be more effective than a single miRNA. However, the key to miRNA's application is overcoming the barrier of delivering miRNA into the cells.

Recently, there have been many tissue engineering strategies that have combined cell-based therapies with scaffolds and different growth factors, morphogens, and differentiation signals to achieve functional tissue-engineered cartilage. The purpose of cartilage tissue engineering is to generate an ECM with biomechanical properties that mimic those of native cartilage. However, stimulating complete regeneration and repair of cartilage has still not been achieved. Studies of miRNAs and their function in regulating expression patterns of different genes in different tissues may revolutionize strategies for cartilage tissue engineering. Combinations of cells with miRNA gene therapy in tissue engineering may bring considerable advances in the treatment of arthritis. Osteogenic differentiation of hMSCs in a three-dimensional scaffold was promoted by an miR-148b mimic and an miR-489 inhibitor [108]. Through overexpressing miR-1 and miR-206 and inhibiting the expression of miR-133, skeletal muscle cell differentiation was improved in three-dimensional bioartificial muscle constructs [109].

## 8. Conclusions and Perspective

As a progressive degenerative joint disorder, OA is characterized by cartilage damage, changes in the subchondral bone, osteophyte formation, muscle weakness, and inflammation of the synovium tissue and tendon. Based on the functions of miRNAs in bone and cartilage, and regulation in the development of OA, miRNAs may provide a novel and efficient method for the regulation of gene expression. Understanding the mechanism of expression and dynamic regulation of miRNAs could be key to promoting chondrogenesis and osteogenesis and to maintaining the balance of bone and cartilage homeostasis to prevent OA progressing, which, in turn, could lead to treating OA directly by preventing tissue degradation and stimulating repair.

It is clear that miRNAs have profound effects on the development of OA, and altered expression of miRNAs in the different stages of OA can be identified* in vitro*, so miRNAs may be useful in the diagnosis of OA as a biomarker. This may be the key to a radical therapy for OA, by regulating the expression of miRNAs. All of these ideas need further advanced studies, more evidence of the networks of miRNAs in arthritis, and more knowledge about gene interference technology* in vivo* to provide new strategies for OA treatment.

Studies on miRNAs have opened a new and exciting vista of gene regulation and offered a new understanding of the mechanisms controlling bone and cartilage development, homeostasis, degradation, and remodeling. Thus, the combination of biological reagents and antiarthritis drugs, together with miRNA treatment, may be more effective and beneficial than traditional treatments. However, the regulation of miRNAs might have carcinogenic effects, given the role of miRNAs as oncogenes. Thus, the side effects of miRNA use in the clinical setting must be considered, and sufficient examinations of miRNA therapy should be performed before clinical use.

## Figures and Tables

**Table 1:** MicroRNA function in bone and cartilage.

Tissue		miRNAs	Target genes	Cell types	References
*Bone*					
Osteoblastogenesis	Upregulated	miR-141/200a	Dlx5	Mouse MC3T3 cells	[13]
		miR-2861	HDAC5	ST2 stromal cells	[14]
		miR-15b	BMPER	hMSCs	[15]
		miR-1228	BMP2K	Human osteoblasts	[16]
		miR-764-5p	CHIP/STUB1	Mouse MC3T3-E1 cells	[17]
		miR-335-5p	DKK1	Mouse preosteoblast cell lines	[18]
		miR-218	SOST, DKK2, SFRP2	Mouse bone marrow stromal cells	[19]
		miR-29a	Osteonectin	Mouse MC3T3 cells	[20]
		miR-27c	Osteonectin	Mouse MC3T3 cells	[20]
		miR-142-3P, miR-27	APC	Hfob1.19 cells	[21, 22]
		miR-210	ACVR1B	Mouse ST2 cells	[23]
		miR-26a	SMAD1	Human ADSCs	[24]
		miR-96	FABP4	hMSCs	[25]
		miR-199a	SOX9, aggrecan	hMSCs	[25]
		miR-140		Bone	[26]
	Downregulated	miR-204/211, miR-133a	Runx2, ALP, Sost	Mouse MC3T3 cells	[13]
		miR-214	Osterix	C2C12 myoblast cells	[27]
		miR-637	Osterix	hMSCs	[28]
		miR-133	Runx2	Mouse C2C12 cells	[29]
		mir-135	Smad5	Mouse C2C12 cells	[29]
		miR-206	Cx43	Mouse C2C12 and osteoblasts	[30]
		miR-100	BMPR2	Human ADSCs	[31]
		miR-155	SOCS1	MC3T3-E1 cells	[32]
		miR-370	BMP-2, Ets1	Mouse MC3T3-E1 cells	[33]
		miR-20a, miR-300	BMP-2	Human ligament fibroblasts	[34]
		miR-135a	Smad5	Mouse mesenchymal cells	[29]
		miR-433	Runx2, ALP	Mouse C3H10T1/2 cells	[35]
		miR-30 (miR-30a, -30b, -30c, -30d)	Smad1, Runx2	MC3T3-E1 cells	[36]
		miR-221, miR-1274a	Wnt signaling	hUSSCs	[37]
		miR-214	ATF4	Mouse MC3T3-E1 cells	[38]
		miR-125b	ErbB2	Mouse MSCs	[39]
		miR-182	FoxO1	C3H10T1/2 MSCs and MC3T3-E1 cells	[40]
		miR-138	PTK2	hMSCs	[41]
		miR-196a	HOXC8	hASCs	[42]
		miR-26a	Smad1	hADSCs	[24]
		miR-378	GalNAc-t7	Hek293 cells	[43]
Osteoclastogenesis	Upregulated	miR-21	PDCD4	Mouse bone marrow-derived monocyte	[44]
		miR-155	SHIP	BMMs	[45]
		miR-34c	Notch1, Notch2, Jag1	Mice osteoblasts	[46]
		miR-223	NF1-A	Mouse bone marrow macrophages	[47]
		miR-378	Caspase-3	Murine RAW264.7 cells	[48]
	Downregulated	miR-29b	c-Fos, MMP-2	DC14+ HPs	[49]
		miR-146a	TRAF6,	Human PBMCs	[50]
		miR-155	SOCS1, MITF	HEK293T cells	[51]
Chondrogenesis	Upregulated	miR-124a, miR-199a	RFX1, HIF1*α*	MSCs	[52]
		miR-140, miR-30a		Mouse MSCs	[53]
		miR-130b, miR-152, miR-28, miR-26b, miR-193b,		hMSCs	[54]
		miR-193b, miR-199a-3p/has-miR-199b-3p, miR-455-3p, miR-210, miR-381, miR-92a, miR-320c, miR-136	C/EBP*β*, RUNX2, BMPR2	hADSCs	[55]
	Downregulated	miR-96	SOX5	MSCs	[52]
		miR-143/-145, miR-132/-212		MSCs	[53]
		miR-145	SOX9	MSCs	[56]
		miR-490-5p, miR-4287, miR-BART8, miR-US25-1	SOX4, SMAD4, BMPR2	hADSCs	[55]
		miR-9	PRTG	MSCs	[57]
		miR-21	GDF-5	Human chondrocytes	[58]
		miR-337	TGFBR2	HEK-293A cells	[59]
OA cartilage	Upregulated	miR-27b	MMP-13	Human chondrocytes	[60]
		miR-483-5p		Human chondrocytes	[61]
		miR-146a	VEGF, Smad4	Human chondrocytes	[62]
		miR-145	SOX9	MSCs	[56, 63]
	Downregulated	miR-140	HDAC4	Human chondrocytes	[64, 65]
		miR-149, miR-582-3P, miR-1227, miR-634, miR-576-5p, miR-641		Human chondrocytes	[61]
		miR-125b	ADAMTS-4	Human chondrocytes	[66]
		mir-127-5P	MMP-13	Human chondrocytes	[67]
		miR-199a	COX-2	Human chondrocytes	[68]
		miR-558	COX-2	Human chondrocytes	[69]
		miR-675	COL II	Human chondrocytes	[70]

## Abbreviations

OA: Osteoarthritis
ECM: Extracellular matrix
miRNAs: MicroRNAs
UTR: Untranslated region
mRISC: miRNA-induced silencing complex
HDAC5: Histone deacetylase 5
BMPER: BMP-binding endothelial regulator
BMP2K: BMP-2 kinase
DKK1: Dickkopf-related protein 1
APC: Adenomatous polyposis coli
ACVR1B: Activin A receptor type 1B
hMSCs: Human mesenchymal stem cells
SOCS1: Suppressor of cytokine signaling 1
hUSSCs: Human unrestricted somatic stem cells
PDCD4: Programmed cell death 4
PBMCs: Peripheral blood mononuclear cells
TRAF6: TNF receptor-associated factor-6
PBMCs: Peripheral blood mononuclear cells.
